# Reaching the top but not feeling on top of the world: Examining women’s internalized power threats

**DOI:** 10.3389/fpsyg.2022.931314

**Published:** 2022-12-15

**Authors:** Sanne Feenstra, Christopher T. Begeny, Jennifer Jordan, Michelle K. Ryan, Janka I. Stoker, Floor A. Rink

**Affiliations:** ^1^Department of Experimental and Applied Psychology, Vrije Universiteit Amsterdam, Amsterdam, Netherlands; ^2^Department of Psychology, University of Exeter, Exeter, United Kingdom; ^3^IMD Business School, Lausanne, Switzerland; ^4^Global Institute for Women’s Leadership, The Australian National University, Canberra, ACT, Australia; ^5^Department of HRM&OB, University of Groningen, Groningen, Netherlands

**Keywords:** power threat, instability, impostor phenomenon, gender, impostor feelings

## Abstract

More and more women are breaking the glass ceiling to obtain positions of power. Yet with this rise, some women experience threats to their power. Here we focus on women’s perceived threats to the stability of their power and the degree to which women feel they do not deserve their power positions, as reflected in their impostor feelings. The present research identifies key workplace characteristics that are associated with these internalized power threats with survey data collected among 185 women in high-power positions. We find that negative workplace experiences (i.e., gender discrimination, denigrating treatment, lack of cultural fit, and lack of mentoring) are associated with a greater sense of power threat, which in turn relates to adverse workplace outcomes (i.e., reduced job satisfaction and increased emotional exhaustion and opting-out intentions). With this unique sample of high-powered women, our findings help illustrate the forces that make women experience power as precarious, thereby shedding light on the disadvantages these women face. We provide suggestions on how to reduce women’s internalized power threats.

## Introduction

More and more women are breaking the glass ceiling to obtain positions of power (ILO, 2020; OECD, 2022). With this rise, it seems vital to fully understand how women experience their power. Although possessing and experiencing power has traditionally been associated with positive outcomes (e.g., less stress, more action and optimism; Anderson and Berdahl, 2002; Keltner et al., 2003), more recent research points to important drawbacks, especially for women (Kark et al., 2021; Vial et al., 2022), and especially when one’s power is threatened (Scheepers et al., 2015). Indeed, research has shown that the fear of losing one’s power and the fear of not deserving one’s power position, that is, impostor feelings or impostor phenomenon (Clance and Imes, 1978), are associated with increased stress (Jordan et al., 2011; Feenstra et al., 2017), anxiety (Cokley et al., 2015), and being distrustful of other people (Mooijman et al., 2019; Feenstra et al., 2020b). These detrimental consequences raise the question of which factors are associated with women’s internalized power threats.

To date, we cannot fully answer this important question. Although much is known about the struggles women face when climbing the power ladder, for example, being discriminated against, excluded from informal networks, lesser feelings of “fitting in,” and lack of mentoring opportunities (Lyness and Thompson, 2000; Peters et al., 2012; Ellemers, 2014; Begeny et al., 2020), we know relatively little about what happens once women obtain such positions of power. Prior research in this realm has focused on how others perceive powerful women (Eagly et al., 2000; Heilman, 2012; Vial et al., 2016; Ellemers, 2018), but scholars have paid less attention to women’s own perceptions and experiences of obtaining positions of power.

**Figure 1:**
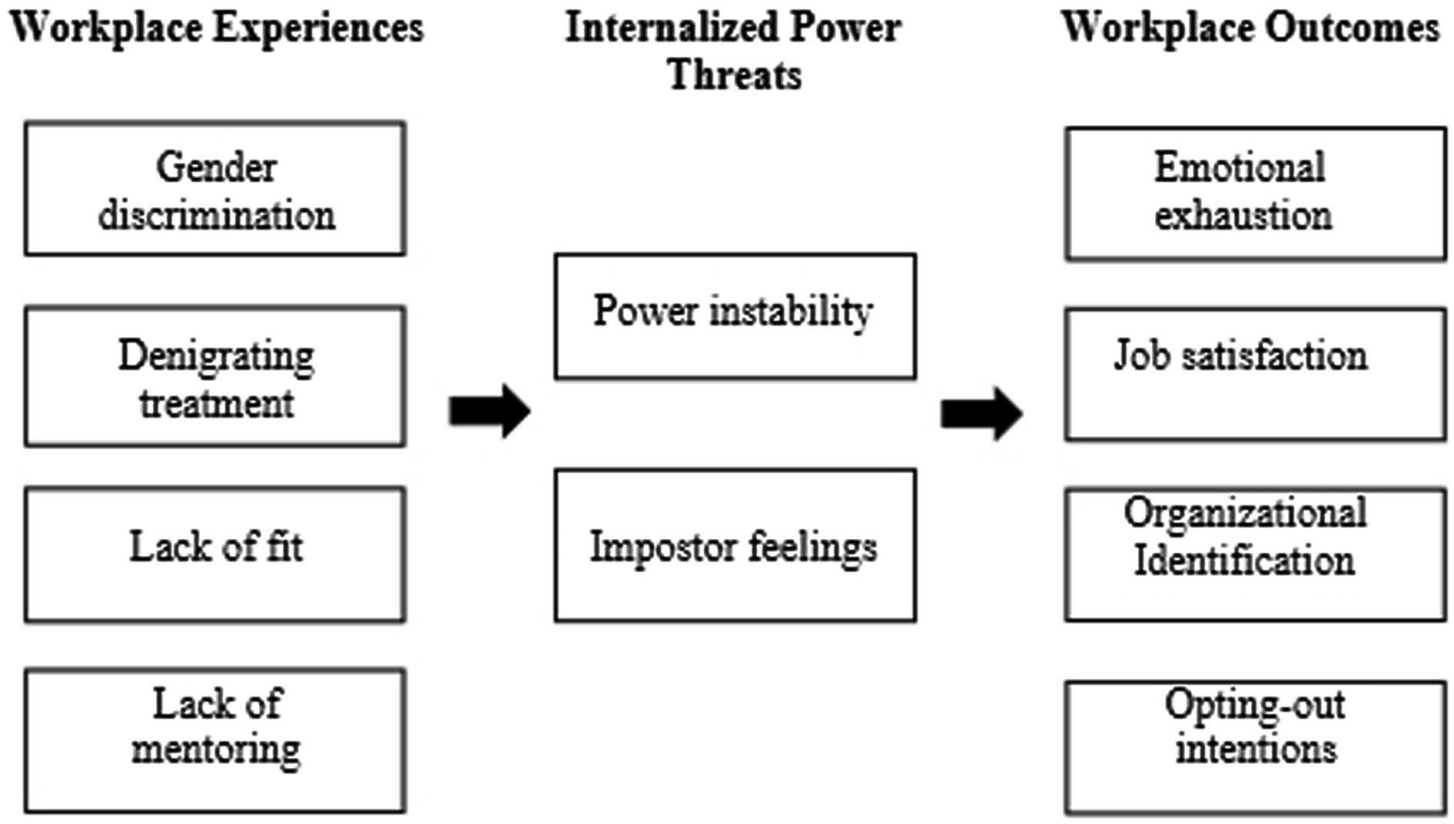
Conceptual model of workplace experiences, internalized power threats, and workplace outcomes. The constructs outlined in the figure are illustrative of the overarching factors of workplace experiences and workplace outcomes.

The present research aims to fill this gap and identifies workplace experiences that are associated with internalized threats to women’s power. In doing so, we build on a wealth of research that has documented specific barriers that women face during their career, and specifically on their paths to power (e.g., Lyness and Thompson, 2000; Hoyt, 2010; Sue, 2010). These workplace experiences include being discriminated against because of one’s gender (Albrecht et al., 2003; Heilman and Caleo, 2018), being interrupted or ‘cut off’ when sharing an idea (Begeny et al., 2021a), being excluded from informal networks (Lyness and Thompson, 2000; Durbin, 2011), and lacking mentoring opportunities (Bogat and Redner, 1985; Lyness and Thompson, 2000). Here we propose that these workplace experiences not only impact women on their paths to power, but also have detrimental consequences once these women reach the top, by shaping their sense of power threat. Thus, our first aim in the present research is to show that the negative workplace experiences of women when climbing the power ladder (i.e., gender discrimination, denigrating treatment, and lack of cultural fit and mentoring) are associated with heightened perceptions of power threat. Our second aim is to show that these internalized power threats, in turn, are related to negative workplace outcomes, such as, reduced job satisfaction and organizational identification, and increased emotional exhaustion and opting-out intentions (see Figure 1).

Overall, the present research contributes to our understanding of how women experience positions of power. While prior research primarily focused on identifying detrimental consequences of external power threats (e.g., Maner and Mead, 2010; Williams, 2014; Neureiter and Traut-Mattausch, 2016), we add to this work by explaining why some women themselves feel that their power is threatened in the first place. In doing so, our work raises important theoretical and practical considerations that could help reduce internalized power threats among women, ultimately making them more comfortable and secure with their power.

## Theory

### The concept of internalized power threats

Power is defined as asymmetric control over valued resources, such that people with higher power control valued resources, while those with lower power are dependent on others for such resources (Magee and Galinsky, 2008). Power is a positive state and therefore powerholders are generally very attentive to potential threats to their power (Williams, 2014). Here, we focus on two types of internalized power threats that women are likely to experience: perceived threats to the stability and deservingness of their power.

First, we focus on stability, that is, the fear of losing power. This type of power threat derives from the fact that power is dynamic. Power relationships can change, such that powerless individuals can climb the power ladder, and the powerful can lose their control over valued resources, descending down the ladder. Being in an unstable power position is stressful, and greatly impacts individuals’ well-being and behavior (Maner and Mead, 2010; Jordan et al., 2011; Scheepers et al., 2015; Feenstra et al., 2017; Feenstra et al., 2020b).

Second, we focus on the extent to which powerholders feel that they do not deserve their power positions. An important phenomenon in this regard is the impostor phenomenon, which refers to feelings that one has received power not because of personal merits or achievements, but due to luck or coincidence. Hence, these individuals tend to feel like impostors and worry that they will be “found out.” This phenomenon was first described in 1978 by clinical psychologists Clance and Imes, who pointed out that high-achieving women were unable to internalize and accept their success and attributed their accomplishment to external factors instead. Although more recent research shows that men can also feel like impostors (Bravata et al., 2019), the phenomenon is more often associated with the experiences of *women* in high-power positions (Meister et al., 2017).

We conceptualize and operationalize these experiences as *internalized* threats to a person’s (in this case women’s) power. In other words, we focus on women’s own perceptions of, and experiences with, power. We further propose that this internalized sense of threat is – at least to some degree – rooted in external (contextually-relevant) factors, including others’ actions toward them. This proposition aligns with other theory and research on group processes, which suggest that how individuals view and think about themselves is shaped by external forces (e.g., others’ actions towards them; for relevant discussions, see, e.g., Huo and Binning, 2008; Feenstra et al., 2020a).

### Workplace experiences and power threats

In the present research, we aim to understand why some women experience threats to their power. Specifically, we build on a wealth of research that has identified specific challenges women face on their paths to power (e.g., Lyness and Thompson, 2000; Hoyt, 2010; Sue, 2010). The first is that women are often discriminated against because of their gender. Compared to men, women are, for instance, evaluated more harshly (Heilman and Caleo, 2018; Begeny et al., 2021b), less likely to be hired for management positions (Gorman, 2005), and paid less for the same work (Bishu and Alkadry, 2017; Catalyst, 2018). Such experiences signal to women that the system is not fair and that important outcomes are beyond their control (Major and Crocker, 1993). This ultimately hurts their psychological well-being (Schmitt et al., 2003). Building on this work, we argue that having been and being subject to discrimination is likely to elicit doubts among women about the continuity of, and their suitability for powerful positions. Overall, we thus propose that women who face gender discrimination on their paths to power will be more likely to feel that their power is threatened.

In addition to overt gender discrimination, women are also likely to encounter more subtle struggles on their paths to power (Lyness and Thompson, 2000). In this regard, research has shown that women are more likely than men to experience denigrating treatment from their colleagues and supervisors, such that they are more likely to be interrupted, criticized, or have their contributions overlooked (for a review see Begeny et al., 2021a) compared to their male counterparts. Such seemingly trivial interactions at work actually communicate that the target is not seen as a person of value or worth and that their insights are valued less compared to that of others (Holleran et al., 2011). Ultimately, such denigrating treatment is important in shaping individuals’ sense of self-worth and potentially, their impostor feelings (Tyler and Blader, 2003; Feenstra et al., 2020a). As such, we argue that being the target of denigrating treatment is likely to be positively associated with experiencing power threats.

Furthermore, previous research demonstrates that women are less likely to feel that they fit in their places of work and report that this lack if fit is an important barrier in climbing the corporate ladder (Lyness and Thompson, 2000; Peters et al., 2012). Research suggests that women are particularly likely to experience such lack of fit in masculine organizational cultures and male-dominated professions (Heilman and Caleo, 2018). Peters et al. (2012), for instance, showed that female trainee surgeons experienced a greater lack of fit with the masculine surgeon prototype than male trainees. Such lack of fit, in turn, causes women to feel out of place and question their own power (Feenstra et al., 2020b; Kark et al., 2021). In corroboration with these arguments, we propose that women’s experiences with a lack of fit are positively associated with experiencing power threats.

Finally, considering the negative experiences of women in the workplace, research suggest that mentoring is essential to women’s career advancement (Tharenou, 2005). Given that women are underrepresented in higher echelons of organizations, however, there are fewer role models and mentoring opportunities available for women who aspire to high-power positions (Bogat and Redner, 1985; Lyness and Thompson, 2000). Research suggest that missing-out on such an important resource of mentoring would likely shake women’s confidence and spur their impostor feelings (Ehrich, 2008; Sanford et al., 2015). As such, we argue that a lack of mentoring opportunities is positively associated with experiencing internalized power threats.

Taken together we thus hypothesize:

*H1:* Negative workplace experiences (i.e., gender discrimination, denigrating treatment, and a lack of fit and mentoring) are positively associated with women’s internalized power threats (i.e., perceptions of power instability and impostor feelings).

### Outcomes of power threats

In a next step, we aim to show that these internalized power threats are associated with detrimental workplace outcomes. First, we build on a wealth of theorizing and empirical support to argue that power threats can harm mental health and shape women’s stress experiences. Indeed, the conservation of resources theory (Hobfoll, 1989) argues that one of the main sources of stress is “when individuals’ resources are threatened with loss” (Hobfoll, 2001, p. 342). In support of such theorizing, empirical studies found that both power instability and impostor feelings are associated with increased stress and anxiety (Sonnak and Towell, 2001; Jordan et al., 2011; Feenstra et al., 2017). Here, we focus on a particularly salient stress experience in the work context, namely burnout. Specifically, we will focus on the core component of burnout which is emotional exhaustion. Emotional exhaustion refers to the experience of feeling “empty” (Maslach et al., 2001; Seidler et al., 2014). Building on the work described above, we argue that women’s internalized power threats are likely to be associated with heightened emotional exhaustion.

In addition to the psychological well-being of female powerholders, other important workplace outcomes that are likely to be related to power threats relate to the enjoyment of, and commitment to, their work. In this domain, researchers have argued that women who experience a lack of career prospects enjoy their work less, are less committed, and more likely to “opt-out” of their organization (Ellemers, 2014). Because of their expectation that they might not get ahead, or are likely to lose their positions and resources, these women are, for instance, less likely to make sacrifices for their work (Meeussen et al., 2021). Consistent with this reasoning, seminal empirical work showed that feelings of impostorism are indeed related to reduced job satisfaction and lack of commitment (Vergauwe et al., 2014; Neureiter and Traut-Mattausch, 2016). Building on this research, we argue that concerns about the stability and deservingness of one’s power are negatively associated with women’s work satisfaction and identification, and positively associated with their intention to “opt-out.” Overall, we hypothesize:

*H2:* Women’s internalized power threats (i.e., perceived power instability and impostor feelings) are positively associated with emotional exhaustion and opting-out intentions, and negatively associated with job satisfaction and organizational identification.

## Materials and methods

You can find more information about our sample, measures, and data analysis in the Supplementary materials here. This study was not preregistered. Participants of this study did not agree for their data to be shared publicly, so supporting data is not pubicly available.

### Participants and procedure

We approached women who were affiliated with an international women’s networking organization that offered leadership development programmes and conferences for its members. We contacted potential participants *via* e-mail to complete an online survey about their experiences in their organizational workplaces. In total 343 potential participants clicked our survey link, of which 241 responded to all key variables (i.e., negative workplace experience and power threat items). Because we focus on women’s experiences of power in this study, we excluded 11 male participants and 45 women who did not occupy a management position^1^. Hence, we conducted our final analysis using 185 women (response rate = 54%; *M*age = 45.45; *SD* = 7.99) from various countries, such as Switzerland (31.9%), United Kingdom (9.2%), and Japan (5.9%). Participants were highly educated, with more than 70% having obtained a Master’s degree or higher. Women reported working in sector such as information technology (16.8%), marketing, sales, and service (8.1%), finance (6.5%), or agriculture, food, and natural resources (7%). Moreover, these women held positions of substantial power in their organizations and institutions, with most of the participants representing either top-management (34.1%) or middle-management positions (54.1%) and supervising up to 20 employees (79%) or more (21%). Though standards for power analyses to test models in SEM are less well-established, the proportion of latent factors to manifest variables specified to test key hypotheses (3 to 10) suggest that we required a sample size of 156 to detect a medium sized effect, or larger (α = 0.05, 1 – *β* = 0.80; conventionally, r of.10/0.30/0.50 is considered small/medium/large effect; Soper, 2022). Overall, this indicates that this study is well powered.

### Measures

Our survey was part of a wider data collection effort. In the Supplementary materials we included a list of additional measurements that were not included here.

#### Workplace experiences

First, we asked participants about negative workplace experiences throughout their career. To measure gender discrimination, we asked participants how often during their career they felt that they were: deprived of certain opportunities (available to others) because of their gender, treated according to stereotypes based on their gender, discriminated against because of their gender, and, viewed negatively because of their gender (Bongiorno et al., 2021; *α* = 0.92). Participants responded to these four items on a scale from 1 (*never*) to 5 (*very often*). We further asked participants to think about the people they interacted with at work (i.e., their co-workers, supervisors, other employees). Following Begeny et al. (2021a) we then measured denigrating treatment by asking how often these people interrupted them or ‘cut them off’ when they were trying to share an opinion or idea, drew attention to relatively minor errors or mistakes they made, seemed to overlook the contributions they made to the organization, and left them out of conversations, group emails, or other informal meetings/gatherings/discussions (*α* = 0.77). Participants responded to these four items on a scale from 1 (*never*) to 5 (*very often*).^2^ Finally, following Lyness and Thompson (2000), we measured lack of fit with three items (*α* = 0.77) and lack of mentoring with four items (*α* = 0.86). We asked participants the extent to which they experienced the following throughout their career: felt pressure to fit in or adapt to the organizational culture, had few role models, felt like they were an outsider (i.e., lack of fit) and not having enough mentoring (e.g., counselling about career opportunities), not having a senior manager who facilitates their career progress, not getting access to the right people (or not knowing the right people), and not receiving enough meaningful feedback about their strengths and weaknesses (i.e., lack of mentoring). Participants responded to these items on a scale from 1 (*not at all*) to 5 (*to a very great extent*).

#### Internalized power threats

We measured power instability by asking participants to indicate the extent to which they felt that their position, status, authority, and power were threatened – a possibility that it will get worse in the future (Feenstra et al., 2020b; *α* = 0.96). Participants responded to all 4 items on a scale from 1 (*not at all*) to 5 (*to a very great extent*). Furthermore, we measured impostor feelings with the 7-item impostorism scale developed by Leary et al. (2000; *α* = 0.92). An example item was: “I’m afraid important people at my work may find out that I’m not as capable as they think I am.”

#### Workplace outcomes

We measured job satisfaction with two items taken from Hackman and Oldham (1980). The items were: “Generally speaking, I am very satisfied with my job” and “I am generally satisfied with the kind of work I do in my job” (*r* = 0.62, *p* < 0.001, *α* = 0.76). We further measured emotional exhaustion with 3-items from the Maslach Burnout Inventory (MBI; Maslach et al., 1996). The items were: “I feel emotionally drained from my work,” “I feel burned out from my work,” and “I feel fatigued when I get up in the morning and have to face another day on the job” (*α* = 0.80). Additionally, we measured organizational identification with six items developed by Mael and Ashforth (1992; *α* = 0.87). An example item is: “My organization’s successes are my successes.” Finally, we measured opting-out intentions by asking participants to what extent they disagreed or agreed with the statement “I often think about quitting my job” (Mobley, 1977). For all outcomes, participants responded on a scale from 1 (*strongly disagree*) to 5 (*strongly agree*).

#### Potential control variables

We considered participants’ age (in years), educational level (1 = did not complete high school, 2 = high school, 3 = some college, 4 = bachelor degree, 5 = master degree, 6 = advanced graduate work or PhD), management level (1 = lower, 2 = medium, 3 = top), number of employees they supervised (1 = no, 2 = 1–5, 3 = 6–10, 4 = 11–15, 5 = 16–20, 6 = more than 20), hierarchical power level (from 1[bottom] to 100 [top]; Lammers et al., 2010), and the gender dominance of the sector in which they worked (dummy coded; dummy 1 [0 = mixed/female-dominated, 1 = male-dominated] and dummy 2 [0 = mixed/male-dominated and 1 = female-dominated]; Mroczek-Dąbrowska and Gaweł, 2020), as potential control variables as previous research suggested that these are associated with our outcome variables (Thompson et al., 2000; Vergauwe et al., 2014; Cokley et al., 2015; Feenstra et al., 2020a; Kark et al., 2021).

## Results

We analyzed our data using IBM SPSS Statistics Version 27 and used SPSS AMOS for structural equation modelling (SEM).

### Factor structure

We conducted confirmatory factor analyses (CFAs) to examine how the data fitted our three-factor model, with workplace experiences, internalized power threats, and workplace outcomes as correlated latent factors and no indicator cross-loadings permitted. Negative workplace experiences was measured by mean scores of gender discrimination, denigrating treatment, and lack of fit and lack of mentoring. Internalized power threat was measured by mean scores of power instability and impostor feelings. Finally, workplace outcomes was measured by mean scores of emotional exhaustion, job satisfaction, organizational identification, and the original score of opting-out intentions. This model showed poor fit to the data (*χ*^2^[32] = 76.75, *p* < 0.001, CFI = 0.91, RMSEA = 0.09, TLI = 0.87), even though all the respective items loaded significantly on their latent variables (all *p* < 0.001). In an effort to improve the fit of the model, we excluded organizational identification from measuring workplace outcomes, as it was the weakest estimate of all latent factors. This three-factor model did show acceptable fit to the data (*χ*^2^[24] = 50.51, *p* = 0.001, CFI = 0.94, RMSEA = 0.08, TLI = 0.91) and all of the respective items loaded significantly on their latent variables (all *p* < 0.001). Furthermore, this three-factor model had a better fit to the data than a one-factor model (in which all constructs loaded on the same latent variable; Δ*χ*^2^ (3) = 69.88, *p* < 0.001), a two-factor model (in which all negative workplace experiences loaded on one factor and power threats and workplace outcomes loaded on one factor; Δ*χ*^2^ (2) = 14.43, *p* < 0.001), a four-factor model (in which the two internalized power threats loaded on separate latent factors; this model was unidentified), and a second-order model (in which the latent factors workplace experiences, internalized power threats, and workplace outcomes were measured by their latent constructs, which we operationalized by their respective items; this model was also unidentified).

### Descriptive statistics

Table 1 reports the descriptive statistics. As expected, all negative workplace experiences (i.e., gender discrimination, negative treatment, lack of fit, lack of mentoring) were positively associated with our two measures of internalized power threats (i.e., power instability and impostor feelings). Furthermore, both internalized power threats were positively associated with emotional exhaustion and opting-out intentions and power instability was negatively associated with job satisfaction. Contrary to expectations, neither of the internalized power threats were associated with organizational identification. Considering these observations and the reduced fit of the model when including organizational identification, we excluded this outcome variable in the main analysis reported below.

**Table 1:** Descriptive statistics.

	*M* (*SD*)	1	2	3	4	5	6	7	8	9	10	11	12	13	14	15	16
1. Age	45. 45 (7.99)	–	–	–	–	–	–	–	–	–	–	–	–	–	–	–	–
2. Educational level	4.70 (0.92)	−0.04	–	–	–	–	–	–	–	–	–	–	–	–	–	–	–
3. Power level	75.11 (15.57)	0.23^**^	0.06	–	–	–	–	–	–	–	–	–	–	–	–	–	–
4. Management level	3.22 (0.64)	0.25^**^	0.07	0.76^**^	–	–	–	–	–	–	–	–	-	–	–	–	–
5. Employee supervision	3.22 (1.76)	0.23^**^	−0.14	0.36^**^	0.29^**^	–	–	–	-	–	–	–	–	–	–	–	–
6. Sector gender dominance (dummy 1)	0.39 (0.49)	0.08	0.03	0.05	0.06	0.13	–	–	–	–	––	–	–	–	-	––	–
7. Sector gender dominance (dummy 2)	0.21 (0.41)	−0.04	0.03	−0.03	0.01	−0.02	−0.41^**^	–	–	–	–	–	–	–	–	–	–
8. Gender discrimination	2.48 (0.97)	0.03	0.27^**^	0.27^**^	0.33^**^	0.05	0.20^*^	−0.10	–	–	–	–	–	–	–	–	
9. Denigrating treatment	2.80 (0.68)	0.04	0.12	0.06	0.18^*^	−0.02	0.03	0.06	0.48^**^	–	–	–	–	–	–	–	-
10. Lack of fit	3.01 (0.96)	0.01	0.27^**^	0.20^*^	0.24^**^	−0.06	0.09	−0.124	0.52^**^	0.43^**^	–	–	–	–	–	–	–
11. Lack of mentoring	2.99 (1.01)	−0.03	0.24^**^	−0.03	0.07	−0.13	0.06	−0.06	0.54^**^	0.36^**^	0.62^**^	–	–	–	–	–	–
12. Impostor feelings	1.66 (0.81)	−0.23^**^	0.11	0.00	0.05	0.04	0.00	−0.07	0.22^**^	0.25^**^	0.34^**^	0.31^**^	–	–	–	–	–
13. Power instability	2.44 (1.15)	0.03	0.12	−0.13	0.03	−0.11	0.12	−0.06	0.31^**^	0.38^**^	0.31^**^	0.32^**^	0.23^**^	–	–	–	–
14. Emotional exhaustion	3.20 (0.85)	0.01	0.11	−0.10	0.04	−0.03	0.10	−0.11	0.29^**^	0.26^**^	0.36^**^	0.23^**^	0.37^**^	0.36^**^	–	–	–
15. Job satisfaction	3.76 (0.88)	0.06	−0.06	0.22^**^	0.18^*^	0.19^*^	−0.07	−0.02	−0.11	−0.25^**^	−0.14^**^	−0.20^**^	−0.09	−0.37^**^	−0.38^**^	–	–
16. Organizational identification	3.80 (0.74)	0.13	−0.04	0.24^**^	0.21^**^	0.13	−0.05	0.05	−0.03	−0.02	−0.17^*^	−0.20^**^	0.01	−0.13	−0.09	0.37^**^	–
17. Opting out	2.67 (1.23)	−0.04	0.00	−0.06	−0.04	0.07	−0.08	0.03	0.18^*^	0.23^**^	0.20^**^	0.24^**^	0.19^**^	0.32^**^	0.46^**^	−0.49^**^	−0.24^**^

N ranges from 154 to 185. ^*^p < 0.05, ^**^p < 0.01. For educational level, 1 = Did not complete high school, 2 = High school, 3 = Some college, 4 = Bachelor degree, 5 = Master degree, 6 = Advanced Graduate work or PhD. For management level, 1 = lower management, 2 = middle management, 3 = top management. For employee supervision, 1 = no, 2 = 1-5, 3 = 6-10, 4 = 11-15, 5 = 16-20, 6 = more than 20. For sector gender dominance dummy 1, 1 = male-dominated, 0 = female-dominated and mixed. For sector gender dominance dummy 2, 1 = female-dominated, 0 = male-dominated and mixed.

### Model testing

**Figure 2:**
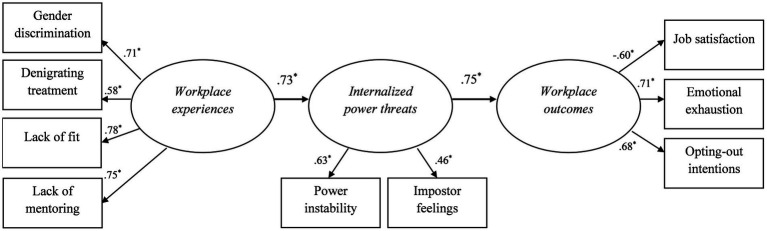
Results for hypothesized model. *N* = 185. *p < 0.05. Standardized regression weights are reported. Model fit: *χ*^2^[25] = 53.93, *p* = 0.001, CFI = 0.93, RMSEA = 0.08, TLI = 0.91.

We conducted structural equation modeling using maximum likelihood method to test our predictions. Specifically, we fitted a three-factor model, with workplace experiences, internalized power threats, and workplace outcomes as latent factors (see Figure 2). We modeled a direct path between workplace experiences and internalized power threats and between internalized power threats and work outcomes. Overall, our path-model showed acceptable fit to the data (*χ*^2^[25] = 53.93, *p* = 0.001, CFI = 0.93, RMSEA = 0.08, TLI = 0.91).^3^ All mean-leveled constructs loaded significantly on their respective latent variable (*p* < 0.001). Moreover, our hypotheses were supported such that negative workplace experiences were positively associated with internalized power threats (estimate = 0.73, *SE* = 0.09, 95% CI [0.55, 0.91]), which in turn were positively associated with workplace outcomes (estimate = 0.75, *SE* = 0.08, 95% CI [0.55, 0.91]). We note that an alternative model with a direct path between workplace experiences and workplace outcomes and, respectively, between workplace outcomes and power threats had poor fit (*χ*^2^[25] = 64.89, *p* < 0.001, CFI = 0.91, RMSEA = 0.09, TLI = 0.87), suggesting our proposed order of variables is also supported. We further note that including a direct path from workplace predictors to workplace outcomes did not significantly improve the fit of the model, Δ*χ*^2^ (1) = 3.42, *p* is between.10 and.05, suggesting that power threats can adequately explain the relationship between workplace predictors and outcomes. Indeed, results showed that internalized power threats mediate the relationships between workplace predictors and outcomes (estimate = 0.54, *SE* = 0.09, 95% CI [0.35, 0.70], *p* = 0.01). Finally, including the control variables, age, educational level, management level, number of employees supervised, and sector dominance did not meaningfully change any of the reported associations (see Supplementary Table 7).

## Discussion

In this investigation, we examined why some women who have reached the top in organizations experience power threats. We demonstrated that negative workplace experiences, such as denigrating treatment, and lack of fit, are positively associated with fears about the stability and legitimacy of women’s power. We further demonstrated that these experiences of internalized power threats are associated with detrimental work outcomes, such as increased emotional exhaustion and intentions to opt-out of one’s organization.

By doing so, our research makes several important contributions. First, our findings provide a better understanding of how women experience positions of high power. While prior research identified obstacles women face on their paths to power (e.g., Eagly et al., 2000; Lyness and Thompson, 2000; Heilman, 2012), we show that negative workplace experiences are associated with women’s perceptions and experiences of positions, once they have reached the top. Although being in a position of power is often associated with positive outcomes, such as status and optimism (Anderson and Galinsky, 2006; Magee and Galinsky, 2008), our findings suggest that for those women who have had negative workplace experiences, positions of power come with specific challenges. Consequently, our work shows that it is important to not just focus on whether women reach the top, but to also look at how women experience these positions of power once they do.

Second, our findings contribute to the work on power threats more generally, and impostor feelings, in particular. Research on power instability, for instance, primarily examined detrimental consequences of unstable power (Jordan et al., 2011; Scheepers et al., 2015; Feenstra et al., 2020b). We contribute to this work by showing the possible origins of such experiences. Furthermore, with regards to impostor feelings, prior research has examined its antecedents, but has tended to focus on *individual* antecedents, such as attachment style or personality of individuals (Bernard et al., 2002; Bravata et al., 2019). Our research takes a different approach, as we adhere to previous calls to examine the role of *context* and the workplace in shaping these experiences (Feenstra et al., 2020a; Kark et al., 2021). In doing so, we contribute to a growing body of work that shows the importance of workplace context in shaping women’s impostor feelings (Muradoglu et al., 2022; Vial et al., 2022).

Although we found support for our theorizing that internalized power threats are associated with reduced job satisfaction, increased emotional exhaustion, and opting out intentions, we found no support that these internalized power threats are associated with a drop in women’s identification with their respective organizations. This might be because being in a position of high power elicits strong organizational identification, even despite the threats that such high-power roles elicit for women. Indeed, prior research has shown that power enhances implicit and explicit role identification (Joshi and Fast, 2013). This explanation should be addressed in future research.

Our research also has important implications for practice. Our findings suggest that to diminish women’s perceptions of power threats, it is important to address the workplace experiences that women regularly face in their careers. Instead of focusing on individual interventions, like trying to boost women’s self-esteem, our results suggest that it is also relevant to take into account the organizational context, and actively focus on (negative) workplace experiences of women. As such, for women to feel more secure with their power, it is important that more structural issues are addressed, such as reducing gender discrimination and denigrating treatment and increasing women’s mentoring opportunities and feelings of fit at work (Ryan, 2022).

This research is not without limitations. Most importantly, the single-source and cross-sectional nature of our data prevents us from identifying cause-and-effect relationships. Hence, it is important that future research replicates our findings using different methods. Researchers could, for example, use multi-sourced data or experimental research designs, manipulating denigrating treatment or lack of fit to test its causal impact on internalized power threats. Furthermore, while our theorizing focused on how women’s past experience shape their current sense of power threat, future research could examine how past *and* present experiences sequentially or simultaneously influence women’s experience of power. In particular, longitudinal research could help tease out how these processes function over time, and can compare women’s career experiences prior to being in positions of power to their experiences when in power. In this regard, future research could, for instance, test a sequential model where women’s negative workplace experiences early in their career shape their sense of power threat, which in turn forms their perceptions of current negative workplace experiences as well.

In addition, while the current research identified a first set of contextual factors that shape women’s power threat, it is important to recognize that other relevant factors could be at play as well, including other external factors (e.g., organizational climate and culture; Kark et al., 2021), internal factors (e.g., lower levels of trait confidence, anxiety), as well as their interplay. In the latter case, one could imagine, for instance, that especially women with initial lower levels of confidence would question their own power when working in a dysfunctional working environment, while women with higher initial confidence would be shielded more from the negative impact of such potential hurtful contexts.

Future research could further expand this work by examining how women’s negative workplace experiences and accompanying power threats impact their leadership behaviours. On the one hand, research in this regard suggests that women’s negative workplace experiences and internalized power threats might give them a leadership advantage. Eagly (2007), for instance, argues that female leaders are more likely to show transformational leadership and go beyond the official requirements of their job because of the stereotypes these women face during their career. Similarly, recent research by Tewfik (2022) suggests that female leaders who feel like impostors are more likely to take the perspective of others, and thus will be more effective leaders. On the other hand, there is research arguing that women’s negative workplace experiences and internalized power threats may sabotage effective leadership. Vial et al. (2016), for instance, propose that female leaders might end up in a “self-reinforcing cycle of illegitimacy” (p. 400) where a lack of validation of their power, results in aggressive leader behaviour. Similarly, research on power instability, suggests that leaders who fear losing their power will be reluctant to share their power and delegate important decisions to their employees (Feenstra et al., 2020b). Additional research is needed to empirically examine these competing predictions.

We further note that while targeting women involved in a women’s international networking organization allowed us access to a hard-to-reach sample of high-powered women, it is possible that women who are affiliated with such an organization have different experiences compared to women who are not members. We could imagine, for instance, that women who experience more gender discrimination or feel more like impostors would be more likely to join such an organization. It is therefore important that future research replicates the reported findings in other representative high-powered, female, and mixed-gender samples.

Finally, our research was limited to women’s experiences with power threats. It would be interesting for future research to examine if, when, and why men feel like their power is under threat and the consequences of this. Probably, men experience less negative workplace experiences and are therefore less likely to experiences these power threats and accompanying negative consequences (Vial et al., 2016). But men might have different contexts in which they are likely to feel like impostors and will likely react more strongly towards threats of their power (Feenstra et al., 2017).

## Conclusion

More and more women are breaking the glass ceiling to obtain positions of power. In this study we have shed light on how these women experience positions of high power. Although traditionally power has been associated with numerous benefits, this research demonstrated that for women with negative workplace experiences, power can also come with particular challenges. Our hope is that this work sparks future research that examines women’s experiences with power and motivates practitioners to create organizational contexts in which high-powered women will feel like they are rightly on top of the world.

## Data availability statement

The raw data supporting the conclusions of this article will be made available by the authors, without undue reservation.

## Ethics statement

Ethical review and approval was not required for the study on human participants in accordance with the local legislation and institutional requirements. The patients/participants provided their written informed consent to participate in this study.

## Author contributions

SF contributed to conceptualization and data collection, and developed questionnaire, analyzing research data, and wrote manuscript. CB and JJ contributed to conceptualization, developing questionnaire and analyzing data, and reviewed and edited writing. MR, JS, and FR contributed to developing questionnaire, conceptualization and reviewed and edited writing. All authors contributed to the article and approved the submitted version.

## Conflict of interest

The authors declare that the research was conducted in the absence of any commercial or financial relationships that could be construed as a potential conflict of interest.

## Publisher’s note

All claims expressed in this article are solely those of the authors and do not necessarily represent those of their affiliated organizations, or those of the publisher, the editors and the reviewers. Any product that may be evaluated in this article, or claim that may be made by its manufacturer, is not guaranteed or endorsed by the publisher.

## Footnotes

^1^We note that conducting the main analysis reported below using a sample including these 45 women in non-management positions, did not meaningfully change any of the reported findings. See Table 6 in the supplementary material for the full results.

^2^For exploratory reasons, we also included a measure of *positive* treatment (3 items; *α* = 0.82; Begeny et al., 2021a). As the present research focuses on the association between *negative* workplace experiences and internalized power threats, we did not include this measure in the main analysis reported below. We note, however, that positive treatment was not associated with power instability (*r* = 0.06, *p* = 0.45), nor impostor feelings (*r* = −0.13, *p* = 0.09). We further note that adding positive treatment as an additional indicator of workplace experiences to the main analysis reported below does not meaningfully change any of the reported findings.

^3^Modification indices suggested that model fit could further be improved by including a direct path from job satisfaction to intentions to opt-out. As this path was not part of our initial investigation, we decided not to include it.
