# Supplementary material for: Reaching the top but not feeling on top of the world: Examining women’s internalized power threats
Source: Front Psychol. 2022 Dec 15;13:931314. doi: 10.3389/fpsyg.2022.931314 (PMC9799960; doi:10.3389/fpsyg.2022.931314)
Supplement: Supplementary file 1 [file Presentation_1.pdf]

Supplementary Information for:

Reaching the Top but not Feeling on Top of the World: Examining Women's Internalized Power Threats

Sanne Feenstra[1]*, Christopher T. Begeny[2], Jennifer Jordan[3], Michelle K. Ryan[2,4], Janka I. Stoker[5], Floor A. Rink[5]

[1]Vrije Universiteit Amsterdam, Department of Experimental and Applied Psychology, Amsterdam, The Netherlands
[2] University of Exeter, Department of Psychology, Exeter, United Kingdom
[3] IMD Business School, Lausanne, Switzerland
[4] Global Institute for Women's Leadership, The Australian National University, Canberra, Australia
[5] University of Groningen, Department of HRM&OB, Groningen, The Netherlands

*** Correspondence:**
Sanne Feenstra
s.feenstra@vu.nl

Includes:
- Measurements
- Sample demographics
- Robustness check for main analysis

Measurements

Measures of negative workplace experiences

The following questions are about your experiences at your work throughout your career.

Gender based discrimination (Bongiorno et al., 2021)
How often at work have you felt that you were…(1-5; Never, Rarely, Sometimes, Often, Very often)
1. deprived of certain opportunities (available to others) because of your gender?
2. treated according to stereotypes because of your gender?
3. discriminated against because of your gender?
4. viewed negatively because of your gender?

Denigrating treatment (Begeny et al., 2021)
Please think for a moment about the people that you have interacted with at work (i.e., your co-workers, supervisors, other employees). How often have they… (1-5; Never, Rarely, Sometimes, Often, Very Often)
1. interrupted you or 'cut you off' when you were trying to share an opinion or idea?
2. drew attention to relatively minor errors or mistakes you had made?
3. seemed to overlook the contributions you made to the organization (things you do, ideas or suggestions you put forward, etc.)?
4. left you out of conversations, group emails, or other informal meetings/gatherings/discussions?

Lack of cultural fit (Lyness & Thompson, 2000)
Please indicate the extent to which you experienced the following throughout your career. (1 = not at all - 5 = to a very great extent)
1. Feeling pressure to fit in or adapt to the organizational culture.
2. Having few role models.
3. Feeling like you are an outsider.

Lack of mentoring (Lyness & Thompson, 2000)
Please indicate the extent to which you experienced the following throughout your career. (1 = not at all - 5 = to a very great extent)
1. Not having enough mentoring (e.g., counseling about career opportunities).
2. Not having a senior manager who facilitates your career progress.
3. Not getting access to the right people (or not knowing the right people).
4. Not receiving enough meaningful feedback about your strengths and weaknesses.

Measures of internalized power threats

Now please think for a moment about your position in your organization. The following questions are about your position in your organization.

Power instability (Morrison, Fast, Ybarra, 2009; Kouchaki & Desai, 2014; Fugate et al., 2008, 2010; Chandler, Kennedy, & Sandhu, 2007)

Please indicate the extent to which you feel that each of the following is *THREATENED - a possibility that it will get worse in the future*?” (1 = not at all threatened - 5 = threatened to a great extent)
1. Your *position* in your organization.
2. Your *status* in your organization.
3. Your *authority* in your organization.
4. Your *power* in your organization.

The following questions are about how you feel at your workplace.

Impostor feelings (Leary, Patton, Orlando, & Funk, 2000)
Please indicate the extent to which the following statements apply to you. (1 = Does not apply to me at all - 5 = Applies to me a lot)
1. Sometimes I am afraid I will be discovered for who I really am at my work.
2. I tend to feel like a phony at work.
3. I'm afraid important people at my work may find out that I'm not as capable as they think I am.
4. In some situations at work, I feel like an imposter.
5. Sometimes I’m afraid others at work will discover how much knowledge or ability I really lack.
6. In some situations in my work I feel like a "great pretender"; that is, I'm not as genuine as others think I am.
7. In some situations in my work, I act like an imposter.

Measures of workplace outcomes

Emotional exhaustion (Maslach, Jackson, & Leiter, 1996)
The following statements are about how you feel at work. Please indicate the extent to which you disagree or agree with the following statements. (1 = Strongly disagree - 5 = Strongly agree)
1. I feel emotionally drained from my work.
2. I feel burned out from my work.
3. I feel fatigued when I get up in the morning and have to face another day on the job.

Job satisfaction (Hackman & Oldham, 1980)
The following statements are about how you feel about your job. Please indicate the extent to which you disagree or agree with the following statements. (1 = Strongly disagree - 5 = Strongly agree)
1. Generally speaking, I am very satisfied with my job.
2. I am generally satisfied with the kind of work I do in my job.

Turnover intentions (Mobley, 1977; 1 = Strongly disagree – 5 = Strongly agree)
TI. I often think of quitting my job.
Organizational identification (Mael & Ashforth, 1992)

The following questions are about how your feel about your organization. Please indicate the extent to which you disagree or agree with the following statements. (1 = Strongly disagree - 5 = Strongly agree)
1. When someone criticizes my organization, it feels like a personal insult.
2. I am very interested in what others think about my organization
3. When I talk about my organization, I usually say ‘‘we’’ rather than ‘‘they.’’
4. My organization’s successes are my successes.
5. When someone praises my organization, it feels like a personal compliment.
6. If a story in the media criticized my organization, I would feel embarrassed.

Measures of demographics and potential control variables

Power (Lammers, Stoker, Stapel, 2010)
1. In your place of work, what level are you in organizational hierarchy? (slider: 0-100)
2. In your position, do you supervise / direct other employees?
    - No
    - 1-5 employees
    - 6-10 employees
    - 10-20 employees
    - More than 20 employees
3. What is your current management position?
    - No management
    - Lower management
    - Middle management
    - Top management)

1. What is your age? (in years)
2. What is your gender?
    - Male
    - Female
3. What is your current country of residence?
4. What is the highest level of education you have obtained?
    - Did Not Complete High School
    - High School
    - Some College
    - Bachelor's Degree
    - Master's Degree
    - Advanced Graduate work or Ph.D.
5. What sector do you work in?
    - Agriculture, Food and Natural Resources
    - Architecture and Construction
    - Arts, Audio/Video Technology and Communications
    - Business Management & Administration
    - Education & Training
    - Finance
    - Government & Public Administration

- Health Science
- Hospitality & Tourism
- Human Services
- Information Technology
- Law, Public Safety, Corrections & Security
- Manufacturing
- Marketing, Sales and Service
- Science, Technology, Engineering & Mathematics

Measures included in the questionnaire (but not in the manuscript)

Positive distinctive treatment (Begeny et al., 2021)
Please think for a moment about the people in your organization (i.e., your co-workers, supervisors, other employees). How often do they… (1 - 5; Never, Rarely, Sometimes, Often, Very Often)
1. ask you for advice?
2. look to you for guidance when they have a question or problem?
3. ask you for help because of certain knowledge, skills or perspectives you have?

Encouragement of feminine values at work
Please indicate to what extent you disagree or agree with the following statements. (1 = strongly disagree - 5 = strongly agree)
1. I feel that feminine values, such as =collaboration, empathy, intuition, integrity, resilience, caring and sharing are welcomed and rewarded at my organization.
2. I feel that the decision-makers in my organization recognize a woman's potential to lead.
3. I feel that integrating feminine qualities, such as emotional intelligence, nurturing and caring is what is needed in organizations, and in the world, right now.

Power legitimacy
Now please indicate to what extent you feel that you DESERVE - are worthy of and qualified for - each of the following in your organization: (1 = not at all deserved - 5 = deserved to a great extent)
1. Your position in your organization.
2. Your status in your organization.
3. Your authority in your organization.
4. Your power in your organization.

Perceived power legitimacy
The following questions are about how you think that other people in your organization (i.e., your co-workers, supervisors, other employees) regard your position in your organization. Please indicate to what extent you feel that other people in your organization think that you DESERVE - are worthy of and qualified for - each of the following. (1 = not at all deserved - 5 = deserved to a great extent)
1. Your *position* in your organization.
2. Your *status* in your organization.
3. Your *authority* in your organization.
4. Your *power* in your organization.

Ambition

Please indicate to what extent you disagree or agree with the following statement. (1 = totally disagree - 5 = totally agree)

1. I am aiming high in this organization (in terms of my goals, ambitions, etc.).
2. Within this organization, I am working hard to advance my professional career.
3. I actively seek out opportunities to take on new endeavors and bigger responsibilities within this organization.
4. I do not have much desire to move up to higher status positions in this organization.

Sample Demographics

Table 1

In your current position, do you supervise/direct other employees? If so, approximately how many?

	Frequency	Percentage
No	31	16.8
Yes, 1-5 employees	51	27.6
Yes, 6-10 employees	35	18.9
Yes, 11-15 employees	22	11.9
Yes, 16-20 employees	7	3.8
Yes, more than 20 employees	39	21.1
Total	185	100

Table 2

What is your current management position?

	Frequency	Percentage
Lower management	22	11.9
Middle management	100	54.1
Top management	63	34.1
Total	185	100

Table 3

What is your current country of residence?

	Frequency	Percentage
Belgium	2	1.1
Brazil	1	0.5
Bulgaria	1	0.5
Cameroon	1	0.5
Canada	1	0.5
Czech Republic	2	1.1
Denmark	2	1.1
Egypt	1	0.5
England / UK	17	9.2
France	9	4.9
Germany	5	2.7
India	1	0.5
Italy	9	4.9
Japan	11	5.9
Luxembourg	3	1.6
Mexico	1	0.5
Nepal	2	1.1
Nigeria	1	0.5
Norway	14	7.6
Portugal	5	2.7
Russia	3	1.6
Singapore	2	1.1
South Africa	1	0.5

Spain	6	3.2
Switzerland	59	31.9
Tanzania	1	0.5
The Netherlands	4	2.2
Turkey	1	0.5
UAE, Abu Dhabi	1	0.5
United States	5	2.7
Missing	13	7.0
Total	185	100

Table 4

What is the highest level of education you have obtained?

	Frequency	Percentage
Did Not Complete High School	1	0.5
High School	9	4.9
Some College	4	2.2
Bachelor's Degree	34	18.4
Master's Degree	112	60.5
Advanced Graduate work or Ph.D.	19	10.3
Missing	6	3.2
Total	185	100.0

Table 5

What sector do you work in?

	Frequency	Percentage	Sector dominance
Agriculture, Food and Natural Resources	13	7.0	Mixed
Architecture and Construction	2	1.1	Male
Arts, Audio/Video Technology and Communications	1	0.5	Mixed / Male
Business Management & Administration	8	4.3	Mixed
Education & Training	8	4.3	Female
Finance	12	6.5	Mixed
Government & Public Administration	3	1.6	Mixed
Health Science	12	6.5	Female
Hospitality & Tourism	3	1.6	Female
Human Services	7	3.8	Female
Informa1tion Technology	31	16.8	Male
Law, Public Safety, Corrections & Security	3	1.6	Male
Manufacturing	11	5.9	Male

Marketing, Sales and Service	15	8.1	Mixed
Science, Technology, Engineering & Mathematics	7	3.8	Male
Transportation, Distribution, and Logistics	3	1.6	Male
Other, please specify	39	21.1	-
Missing	7	3.8	-
Total	185	100	-

Robustness Check for Main Analysis

Table 6

Results for Hypothesized Model Based on Full Sample (Including Non-Managers)

	Estimate	SE	95% Confidence Interval Lower bound	95% Confidence Interval Upper bound
Gender discrimination ⟵ Workplace experiences	.70*	.05	.60	.79
Denigrating treatment ⟵ Workplace experiences	.61*	.06	.49	.73
Lack of fit ⟵ Workplace experiences	.80*	.04	.72	.86
Lack of mentoring ⟵ Workplace experiences	.75*	.04	.67	.82
Power instability ⟵ Internalized power threat	.62*	.07	.50	.75
Impostor feelings ⟵ Internalized power threat	.55*	.07	.40	.67
Job satisfaction ⟵ Workplace outcomes	-.60*	.07	-.72	-.46
Emotional exhausation ⟵ Workplace outcomes	.70*	.07	.54	.83
Opting-out intentions ⟵ Workplace outcomes	.67*	.08	.53	.82
Workplace experiences ⟶ Internalized power threats	.75*	.08	.57	.88
Internalized power threats ⟶ Workplace outcomes	.80*	.08	.62	.96
Chi^2 (df)	65.27 (25)			
RMSEA	.09			
CFI	.93			
TLI	.90			

Notes. $N = 226$. * $p < .05$. Estimates are standardized regression weights.

Table 7

Results for Hypothesized Model including Control Variables

	Estimate	SE	95% Confidence Interval Lower bound	Upper bound
Age ⟵ Internalized power threats	-.05	.17	-.44	.26
Educational level ⟵ Internalized power threats	-.07	.10	-.30	.11
Management level ⟵ Internalized power threats	.08	.18	-.26	.50
# employees supervised⟵ Internalized power threats	.07	.12	-.17	.32
Hierarchical power level⟵ Internalized power threats	-.37	.19	-.74	.04
Sector gender dominance (dummy 1) ⟵ Internalized power threats	.08	.11	-.14	.28
Sector gender dominance (dummy 2) ⟵ Internalized power threats	.07	.11	-.15	.30
Age ⟵ Workplace outcomes	.01	.17	-.36	.39
Educational level ⟵ Workplace outcomes	-.03	.09	-.21	.15
Management level ⟵ Workplace outcomes	-.31	.20	-.73	.08
# employees supervised ⟵Workplace outcomes	.03	.13	-.20	.31
Hierarchical power level⟵ Workplace outcomes	.24	.22	-.14	.67
Sector gender dominance (dummy 1)⟵ Worplace outcomes	-.12	.12	-.36	.11
Sector gender dominance (dummy 2) ⟵ Workplace outcomes	-.03	.12	-.28	.15
Gender discrimination ⟵Workplace experiences	.71*	.07	.56	.83
Denigrating treatment ⟵ Workplace experiences	.53*	.09	.32	.67
Lack of fit ⟵ Workplace experiences	.76*	.06	.62	.87
Lack of mentoring ⟵ Workplace experiences	.71*	.06	.58	.80
Power instability⟵ Internalized power threat	.65	.12	.39	.88
Impostor feelings ⟵Internalized power threat	.40*	.14	.12	.70
Job satisfaction⟵Workplace outcomes	-.62*	.09	-.80	-.44
Emotional exhausation⟵ Workplace outcomes	.63*	.09	.41	.78
Opting-out intentions⟵Workplace outcomes	.71*	.10	.48	.89
Workplace experiences ⟶ Internalized power threats	.72*	.12	.46	.97
Internalized power threats ⟶ Workplace outcomes	.85*	.12	.63	1.08
Chi^2 (df)	127.91 (67)			
RMSEA	.08			
CFI	.89			
TLI	.81			

Notes. N = 154. * *p* < .05. Estimates are standardized regression weights.
